# Endoscopic ultrasound-guided transmural drainage for subphrenic abscess: report of two cases and a literature review

**DOI:** 10.1186/s12876-018-0782-2

**Published:** 2018-04-27

**Authors:** Shinichi Morita, Kenya Kamimura, Takeshi Suda, Chiyumi Oda, Takahiro Hoshi, Tsutomu Kanefuji, Kazuyoshi Yagi, Shuji Terai

**Affiliations:** 1 Department of Gastroenterology and Hepatology, Uonuma institute of Community Medicine Niigata University Hospital, 4132 Urasa, Minamiuonuma City, Niigata 949-7302 Japan; 2 Division of Gastroenterology and Hepatology, Graduate School of Medical and Dental Sciences, Niigata University, 1-757 Asahimachido-ri, Chuo-ku, Niigata 951-8510 Japan

**Keywords:** Endoscopic ultrasound-guided transmural drainage, Subphrenic abscess, Intra-abdominal abscess, EUS

## Abstract

**Background:**

An intra-abdominal abscess can sometimes become serious and difficult to treat. The current standard treatment strategy for intra-abdominal abscess is percutaneous imaging-guided drainage. However, in cases of subphrenic abscess, it is important to avoid passing the drainage route through the thoracic cavity, as this can lead to respiratory complications. The spread of intervention techniques involving endoscopic ultrasonography (EUS) has made it possible to perform drainage via the transmural route.

**Case presentation:**

We describe two cases of subphrenic abscess that occurred after intra-abdominal surgery. Both were treated successfully by EUS-guided transmural drainage (EUS-TD) without severe complications. Our experience of these cases and a review of the literature suggest that the drainage catheters should be placed both internally and externally together into the abscess cavity. In previous cases there were no adverse events except for one case of mediastinitis and pneumothorax resulting from transesophageal drainage. Therefore, we consider that the transesophageal route should be avoided if possible.

**Conclusions:**

Although further studies are necessary, our present two cases and a literature review suggest that EUS-TD is feasible and effective for subphrenic abscess, and not inferior to other treatments. We anticipate that this report will be of help to physicians when considering the drainage procedure for this condition. As there have been no comparative studies to date, a prospective study involving a large number of patients will be necessary to determine the therapeutic options for such cases.

## Background

Intra-abdominal abscess is a severe infectious condition refractory to cure with antibiotic monotherapy [1, 2]. Therefore, the basic treatment strategy has been percutaneous imaging-guided drainage [3, 4]. However, in cases of subphrenic abscess [5], the lesion is surrounded by other organs such as the lungs, liver and intestines, sometimes making it difficult to secure a safe drainage route percutaneously, and surgical drainage may therefore be needed, even though it is invasive [6, 7].

Recently, the development of endoscopic ultrasonography (EUS) intervention techniques has made it possible to perform puncture and drainage via the transmural route for the deeper part of the abscess, where percutaneous treatment has been difficult [8–13]. Here we report two cases of subphrenic abscess that were treated successfully with EUS-guided transmural drainage (EUS-TD). With a view to developing a safe and effective drainage procedure, we reviewed 14 reported cases of subphrenic abscess treated with EUS-TD in addition to our own two cases. We anticipated that the information in this report would be of help to physicians for safer and more effective management of subphrenic abscesses.

## Case presentation

### Case 1

**Fig. 1:**
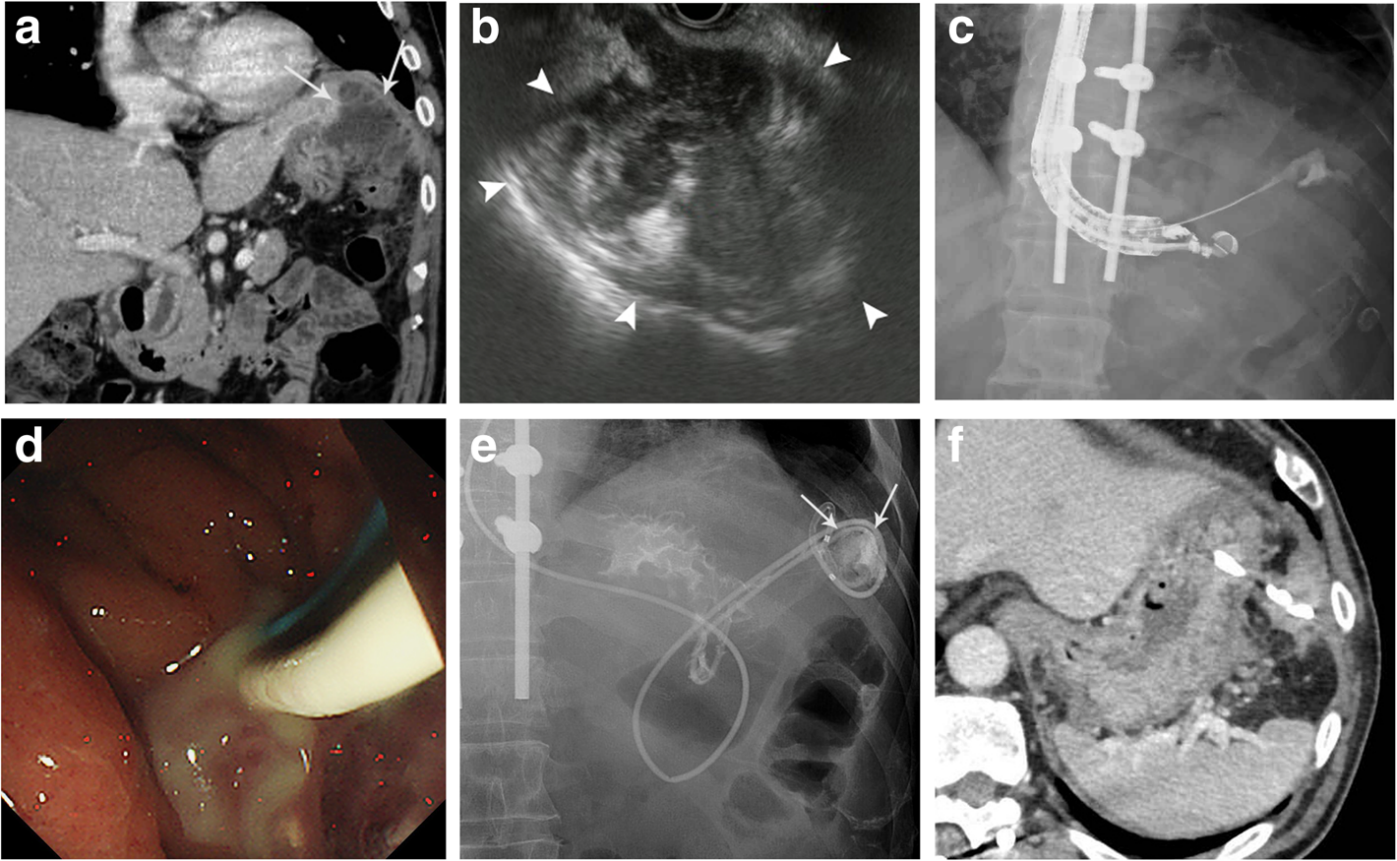
**a** Contrast-enhanced computed tomography (CT) shows fluid collection (arrows) in the left subphrenic area adjacent to the fornix of the stomach. **b** Endoscopic ultrasound shows irregular fluid collection (arrowheads). **c** Fluoroscopic image shows a 19-gauge needle inserted into the abscess cavity. **d** Endoscopic image shows much viscous pus extruding into the stomach through the stents. **e** Fluoroscopic image with contrast medium enhancement via the naso-abscess external catheter shows shrinkage of the abscess cavity (arrows) and no leakage from the anastomosis of the transected colon. **f** CT reveals that the drainage stent and that the drained abscess cavity became shrank

A 66-year-old Japanese man underwent laparoscopic partial resection for sigmoid colon cancer. His progress after surgery was smooth and he was discharged on the 7th postoperative day. However, 17 days after surgery he developed a high-grade fever of 40 °C and upper abdominal pain, necessitating readmission to our hospital. Laboratory examinations demonstrated a significant increase in the white blood cell count to 16,000/μL and a C-reactive protein level of 19.2 mg/dL. Contrast-enhanced computed tomography (CT) revealed an encapsulated mass of accumulated fluid 90 mm in diameter adjacent to the upper part of the stomach **(**Fig. 1a, b**)**. On the basis of the symptoms, and the findings of laboratory and imaging examinations, a diagnosis of left-sided subphrenic abscess was made. Tazobactam/piperacillin, a broad-spectrum antibiotic, was administered three times daily (4.5 g/day) for 4 days, but little improvement was observed. Therefore, a decision was made to perform drainage of the abscess. As the percutaneous drainage line showed an intrathoracic pathway, and a clear image was obtained via the stomach by EUS, EUS-TD was selected as the drainage method.

A linear-array echoendoscope (GF-UCT260 and UE-ME2 US system; Olympus Medical, Tokyo, Japan) was employed for the procedure and EUS-images were carefully checked to examine whether the abscess wall was well matured. It showed that the wall was thick enough and well adherent to the stomach wall. Therefore, following confirmation of a clear puncture line using color Doppler, as standard technical method, a 19-gauge needle (SonoTip pro control®, Medi-Globe, Rosenheim, Germany) was inserted into the abscess, confirming aspiration of pus **(**Fig. 1c**)**. Then, a 0.025-in. guidewire (Visiglide 2™; Olympus Medical) was inserted through the needle followed by insertion of a cautery catheter (Cyst-Gastro set®; Century Medical, Seoul, Korea) into the cavity, creating a ostomy to the abscess from the stomach under endosonographic and fluoroscopic guidance. After dilation of this ostomy using an 6-mm-diameter balloon catheter (ZARA®; Century Medical), a 5-cm-long 7F double pigtail plastic stent (Advanix®; Boston Scientific, Marlborough, USA) and 6F naso-abscess catheter (Quick Place V™; Olympus Medical) were inserted to drain the pus either into the stomach and extracorporeally **(**Fig. 1d**)**. Culture of the pus demonstrated infection with *Klebsiella pneumoniae*, and on the day after drainage the high fever and abdominal pain were improved. As the waste liquid was high-viscosity pus, the naso-abscess catheter was irrigated with physiological saline to avoid any obstruction. Fluoroscopic imaging with contrast medium enhancement from the naso-abscess catheter demonstrated shrinkage of the abscess cavity and no leakage at the anastomosis of the transected colon (Fig. 1e). Therefore, the naso-abscess catheter was removed after 7 days and disappearance of the abscess was confirmed 1 month later **(**Fig. 1f**)**, followed by the removal of the pigtail stent. The post-procedure course was uneventful and adjuvant chemotherapy was performed successfully.

**Fig. 2:**
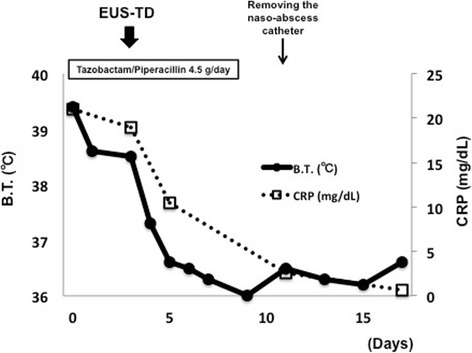
Changes in body temperature and C-reactive protein level after EUS-TD in Case 1

To date, no recurrence of the abscess or metastases from the colon cancer has been observed. The clinical course including changes in temperature and the serum C-reactive protein level are shown in Fig. 2.

### Case 2

**Fig. 3:**
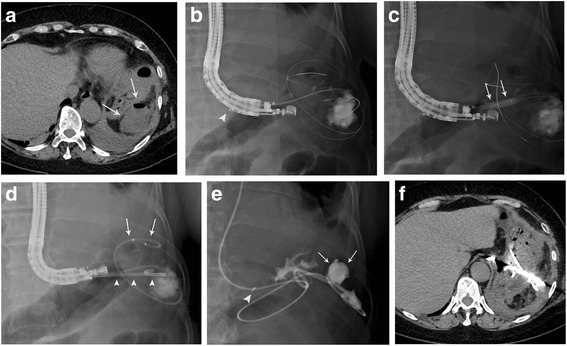
**a** CT shows accumulated fluid containing gas (arrows) in the left subphrenic area adjacent to the fornix of the stomach. **b** Fluoroscopy image shows the guidewires coiling in the abscess cavity. The hemoclip (arrowhead) is placed at the esophageal junction to avoid transesophageal puncture. **c** Fluoroscopic image shows an 8-mm-diameter balloon catheter (arrows) dilating the ostomy between the stomach and the abscess cavity. **d** A 5-cm-long 7F double pigtail stent (arrowheads) and a 6F naso-abscess catheter (arrows) are placed into the cavity. **e** Fluoroscopic image with contrast medium enhancement via the naso-abscess external catheter shows shrinkage of the abscess cavity (arrows). The hemoclip (arrowhead) is placed at the esophageal junction. **f** CT demonstrates the drainage catheter and stent, and reduction of the abscess cavity

**Fig. 4:**
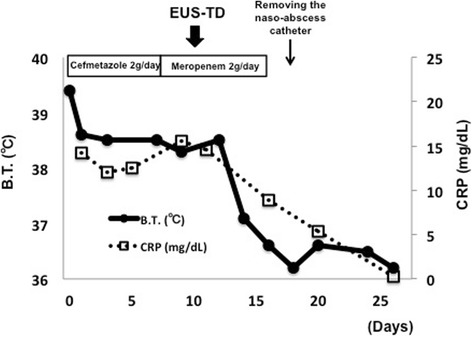
Changes in body temperature and C-reactive protein level after EUS-TD in Case 2

A 57-year-old Japanese woman with diabetes mellitus underwent right hemicolectomy for cancer of the ascending colon. She suffered from a persistent high-grade fever of 40 °C for 14 days after surgery, and despite antbiotic therapy she showed no improvement. Laboratory examinations demonstrated a significant increase in the white blood cell count to 13,300/μL and the C-reactive protein level to 14.6 mg/dL. CT imaging revealed a mass of accumulated fluid 70 mm in diameter containing gas, suggesting formation of an abscess adjacent to the upper part of the stomach **(**Fig. 3a**)**. Based on these findings, a diagnosis of subphrenic abscess was made and cefmetazole was administered twice daily (2 g/day) for 7 days. However, as the effect was insufficient, the antibiotic was changed to meropenem, a broad-spectrum antibiotic, twice daily (2 g/day) for 5 days. As this in turn proved ineffective, EUS-TD was performed because no appropriate percutaneous puncture line was available. EUS-image showed the maturation of abscess wall with its thickness and adherence to the stomach wall. Therefore, a 5-cm-long 7F double pigtail plastic stent and a 6F naso-abscess catheter were placed into the abscess cavity (Fig. 3b, c, d), as described for Case 1. Gram staining and culture of a sample of the aspirate revealed *Escherichia coli*. On the day after drainage, the high fever ameliorated, and the naso-abscess catheter was irrigated with physiological saline for several days. After 7 days, fluoroscopic imaging with contrast medium enhancement via the naso-abscess catheter showed shrinkage of the abscess cavity (Fig. 3e), and therefore the catheter was removed. CT at 1 month after drainage showed disappearance of the abscess cavities (Fig. 3f), so the pigtail stent was removed with an endoscope. For 7 months thereafter, there was no evidence of abscess recurrence. The clinical course including changes in temperature and the serum C-reactive protein level are shown in Fig. 4.

## Discussion and conclusions

Intra-abdominal abscesses often occur following intra-abdominal surgery, trauma, severe enteritis, perforation of the intestine, and acute pancreatitis [5]. If there is any delay in diagnosis and treatment, sepsis may develop, followed by septic shock with a potentially fatal outcome [1, 2]. Therefore, prompt and adequate treatment is needed. The basic treatment for abscess is drainage, and the first choice of drainage route is percutaneous [3, 4, 14]. Imaging-guided percutaneous abscess drainage has been shown to be effective, and has been a great milestone in the treatment of abscesses [3, 14–18]. The modalities used for this procedure have been mostly ultrasonography and computed tomography. Previous reports of percutaneous drainage for intra-abdominal abscess have documented success rates of between 60% and 92% [3, 15–18]. In series with high success rates, imaging-guided percutaneous abscess drainage was performed when an available access route could be secured. However, when the abscess cavity is deeply located in the body, it may be difficult to secure a drainage route due to the surrounding organs. Therefore, for such cases, surgical drainage has remained a standard [3, 6, 17], even though it is highly invasive [3]. With the growing number of elderly patients worldwide, who cannot tolerate highly invasive techniques, there has been a need to develop a less invasive method for drainage of deeply seated abscesses.

Subphrenic abscess is characterized by accumulation of pus or infected fluid in the space between the diaphragm, liver, spleen and intestines [5]. This makes it difficult to puncture while avoiding other organs during intra-abdominal passage, and the drainage route often passes through the thoracic cavity [7, 19, 20]. However, an intrathoracic route carries a risk of severe complications such as pleural effusion, pneumothorax, pyothorax and mediastinitis [7, 14, 19, 21]. To avoid such complications, the transmural drainage route has been tested with the development of EUS. Since the first report of EUS-TD for pancreatic pseudocyst [22], the technique has been widely used [10, 13, 23–25]. EUS-TD has a number of advantages: 1) excellent visualization of the cavity of the intra-abdominal abscess, even when located deeply; 2) the Doppler mode is able to demonstrate the puncture line, thus avoiding major vessels; 3) real-time US images can be obtained during the procedure. To date, 14 cases, including our case, of subphrenic abscess treated with EUS-TD have been reported, and their details are summarized in Table 1 [8–10, 13, 26–28].

**Table 1:** Summary of EUS-TD for intra-abdominal abscess

Number	Age (year)	Gender	Primary disease	Etiology	Abscess location	Size of maximum axis (mm)	EUS-TD route	Drainage modality	Time to removal of external drainage	Time to removal of internal stent	Complications	Recurrence	Reference
1	64	M	Chronic renal failure	Abdominal inflammation	Left	55	Transgastric	7F NA catheter,	8 days	4 weeks	None	None	[8]
								10F DP stent					
2	40	F	GIST	Surgery	Left	50	Transgastric	7F NA catheter,	8 days	4 weeks	None	None	[8]
								10F DP stent					
3	47	M	Acute pancreatitis	Abdominal inflammation	Left (spleen)	90	Transgastric	8.5F NA catheter,	1 day	3 months	None	None	[26]
								Two 10F DP stents					
4	59	M	Liver metastases of rectal cancer	Surgery	Right	50	Transgastric	Two 10F DP stents	None	3 weeks	None	None	[9]
5	36	F	Chronic pancreatitis	Abdominal inflammation	N/A	N/A	Transesophageal	Two 10F DP stents	None	3 months	Mediastinitis, Pneumothorax	None	[10]
6	60	M	Myasthenia gravis	Abdominal inflammation	N/A	200	Transesophageal	Two 10F DP stents	None	No removal	None	None	[10]
7	54	F	None	Trauma	Left	66	Transgastric	A 10F DP stent	None	2 weeks	None	None	[10]
8	44	M	Ulcerative colitis	Surgery	Left	85	Transgastric	7F NA catheter,	2 days	6 weeks	None	None	[10]
								Two 10F DP stents					
9	60	M	Liver cirrhosis	Immunosuppressant	Left	51	Transgastric	7F DP stent	None	2 weeks	None	None	[27]
10	57	M	Colon cancer	Surgery	Left	100	Transgastric	Two 10F DP stents	None	No removal	None	None	[28]
11	60	F	Rectal cancer	Surgery	Left	61	Transgastric	6F NA catheter	11 days	None	None	None	[13]
12	69	M	IPMN	Surgery	Left	70	Transgastric	6F NA catheter,	10 days	No removal	None	None	[13]
								7F DP stent					
13	66	M	Colon cancer	Surgery	Left	93	Transgastric	6F NA catheter,	7 days	2 months	None	None	Our case 1
								7F DP stent					
14	57	F	Colon cancer	Surgery	Left	67	Transgastric	6F NA catheter,	7 days	3 months	None	None	Our case 2
								7F DP stent					

*EUS-TD* endoscopic ultrasound-guided transmural drainage, *M* male, *F* female, *GIST* gastrointestinal stromal tumor, *IPMN* intraductal papillary mucinous neoplasm, *N/A* information not available

*F* French, *NA* catheter, naso-abscess catheter, *DP stent*, double pigtail stent

The reported technical and clinical success rates were both 100% (14/14). The drainable abscess location was reported both left and right sides under the diaphragm. However, because of the anatomical location of the stomach, left-sided drainage via the transgastric route is preferable (92.9%; 13/14). The organs through which the route passes include not only the stomach but also the esophagus (14.3%; 2/14), when the abscess location is adjacent to the intestinal tract.

In both of the present cases, the EUS image was informative to confirm the maturity of the abscess wall and its adherence to the stomach wall in both of our cases. Therefore, especially when a drainage is considered in cases of recently developed abscess, the careful consideration of the conditions and the modification of techniques, devices, including balloon catheters, stent, and etc. are necessary. Drainage methods can be external, internal, or both, as was the case in our patients. Our review indicated that in 50% (7/14) of cases, both an external naso-abscess drainage catheter and an internal drainage stent were placed. There are several advantages using two such drains. Through a naso-abscess catheter can be aspirated as much of the pus as possible immediately after placement and allow identification of any causative microbial agents. In addition, irrigation of an abscess cavity with a saline can prevent a development of pus obstruction of a catheter by highly viscous pus. It also allows contrast medium enhancement to confirm the size of the abscess cavity and postoperative anastomotic leakage if necessary. An internal drainage stent maintains the transmural drainage tract and allows exchange or addition of new drainage stents. However, naso-abscess catheter placement decreases the patient’s quality of life due to nasopharyngeal discomfort. The median period until removal of the naso-abscess catheter was 7 days in the cases we reviewed. Whereas Kassi et al. reported that the median time until removal of the catheter for percutaneous intra-abscess drainage was 13.2 days [4]. On the other hand, 43% (6/14) of presented cases were improved with internal drainage only. Five of these six cases were treated by placement of two 10F DP stents. The wide-lumen stent can secure a sufficient drainage and has reliable long-term patency. Moreover, by means of multiple stenting, pus outflow occurs not only through the lumina of the stents, but also through the space between the stents. If placement a naso-abscess catheter is avoided, it is preferable to place multiple larger-diameter stents. However, it remains to be determined whether internal drainage using multiple stents or external drainage with a naso-abscess catheter is more effective.

Complications of EUS-TD for treatment of pancreatic fluid collections, that can be considered to be similar to that of subphrenic abscess, have been reported in about 10% of cases, and involve mainly bleeding and perforation [29–33]. Our literature review revealed one complication associated with EUS-TD for subphrenic abscess (1/14), in a patient treated via the transesophageal drainage route who developed mediastinitis and pneumothorax. These complications have also been reported for EUS-fine needle aspiration and EUS-TD via the esophagus via the transthoracic route [10, 34–36]. Therefore, the transesophageal route should be avoided if possible, and some form of percutaneous or surgical procedure may have to be considered for such cases. We have recently reported a useful technique of clip anchoring at the esophagogastric junction [37]. Hemoclips were placed at the esophageal junction in advance, and the drainage catheters were inserted into the abscess cavity under fluoroscopic observation at a point distal to the clips. This technique can help to avoid intrathoracic passage.

Our review indicated that 42.9% (6/14) of patients were over 60 years old. As the number of elderly patients is increasing worldwide, less invasive and more effective methods for abscess drainage are needed. Recently in the EUS-intervention field, reports of a use of lumen apposing metal stents (LAMS) have been increasing [38, 39] and such stents have actually been used for drainage of pancreatic fluid collections and abdominopelvic abscesses [34]. The wide lumen allows effective drainage and the characteristic stent edges prevent leakage into the abdominal cavity and stent migration. It is expected that this type of stent may be placed more safely, and allow more effective drainage than an ordinary stent. On the other hand, there have been some reports of severe adverse events such as perforation and massive bleeding related to EUS-TD with LAMS for pancreatic walled-off necrosis [33]. Therefore, further prospective studies are essential to prove its usefulness. Our present experiences and literature review indicate that EUS-TD for subphrenic abscess is feasible and effective. It is anticipated that the information summarized in this article may be of help to physicians when considering the drainage procedure for this condition. As there have been no comparative studies to date, a prospective study involving a large number of patients will be necessary to determine the therapeutic options for individual cases.

## Abbreviations

BT: body temperature
CRP: C-reactive protein
CT: computed tomography
DP stent: double pigtail stent
EUS: Endoscopic ultrasonography
EUS-TD: Endoscopic ultrasound-guided transmural drainage
F: French
LAMS: lumen apposing metal stent
NA catheter: naso-abscess catheter
